# The relational foundation of self-determined drive in adolescent athletes: linking perceived relationship with parents to autonomous motivation

**DOI:** 10.3389/fpsyg.2026.1875168

**Published:** 2026-08-05

**Authors:** Inwoo Kim, Sujin Kim

**Affiliations:** Department of Sports Science Convergence, Dongguk University, Seoul, Republic of Korea

**Keywords:** athletic identity, autonomous motivation, elite adolescent athletes, parental relationship, self-esteem, sequential mediation

## Abstract

**Background:**

The motivational orientation of adolescent athletes is a primary determinant of their long-term trajectories, yet the precise mechanisms through which the relational foundation provided by parents is internalized into a self-determined drive remain underexplored.

**Objective:**

This study investigated the psychological pathways between the perceived relationship with parents and autonomous motivation, specifically examining the sequential mediating roles of global self-esteem and domain-specific athletic identity among elite youth soccer players.

**Methods:**

Data were collected from 237 elite male high school soccer players affiliated with the Korea Football Association (KFA). Structural equation modeling (SEM) was employed to test the direct and indirect associations within the hypothesized serial mediation framework, and a bootstrapping procedure with phantom variable modeling was used to isolate specific indirect effects.

**Results:**

Perceived relationship with parents significantly and positively was associated with both self-esteem and autonomous motivation. Although parental influence did not directly predict athletic identity, it exhibited a significant indirect effect through the mediation of self-esteem. Furthermore, the sequential mediation analysis confirmed a significant serial path: a positive perception of the parental relationship was first linked to the athlete’s foundational self-esteem, which in turn could support a robust athletic identity, ultimately associating with higher levels of autonomous motivation.

**Conclusion:**

For external familial support to be effectively linked to a self-determined motivational orientation, it may need to be associated with global self-esteem and specialized role-identification. These findings suggest that practitioners and parents should prioritize the cultivation of an athlete’s global self-worth as a prerequisite for developing a resilient athletic identity and sustaining high-quality motivation in competitive sports environments.

## 1 Introduction

Within the high-stakes social ecosystem of youth sports, elite adolescent student-athletes constantly navigate intense and highly evaluative environments. In this competitive context, the motivational orientation of adolescent athletes plays a pivotal role in shaping their long-term trajectories, encompassing both athletic excellence and holistic psychosocial development (Teixeira et al., 2012; Martins et al., 2017). Beyond mere performance outcomes, the nature of an athlete’s drive is closely linked to sustained persistence in competitive environments while simultaneously fostering emotional maturity and subjective well-being (Martins et al., 2017). According to Self-Determination Theory (SDT), the qualitative dimension of motivation carries greater weight than its overall intensity (Deci and Ryan, 2000, 2008). Specifically, autonomous motivation defined by actions rooted in a sense of volition, personal value, and choice functions as a fundamental determinant for long-term engagement and adaptive psychological health (Deci and Ryan, 2008; Amorose et al., 2016). Conversely, when motivation lacks this self-determined quality, athletes often experience diminished functioning and an increased risk of burnout (Lienhart et al., 2020; O’Rourke et al., 2012).

Given these profound implications, a primary objective in contemporary sport psychology has been to identify the specific social and environmental antecedents associated with such high-quality motivational states in elite youth populations (Castillo-Jiménez et al., 2022). Parents function as the primary socializers who fundamentally shape an athlete’s experience throughout childhood and adolescence (Harwood and Knight, 2015). By providing emotional, organizational, and financial resources, they exert a profound influence on the motivational frameworks and self-perceptions of student-athletes (Fredricks and Eccles, 2004; Wolfenden and Holt, 2005). Recent empirical evidence suggests that the impact of the familial environment may even outweigh that of the coaching staff in certain psychological domains; specifically, parent-initiated mastery climates have proven to be potent correlates of an individual’s self-esteem and autonomous regulation (O’Rourke et al., 2014). The impact of parental involvement, however, is not uniformly beneficial, as the qualitative nature of the parent-child relationship serves as a primary contingency, where excessive expectations can transition into severe psychological stressors (Amado et al., 2015; Schwebel et al., 2016).

Self-esteem emerges as a primary psychological mediator in this internalization process, conceptualized as an individual’s subjective and global evaluation of self-worth (Rosenberg, 1965). Adolescents construct this sense of self-regard by internalizing appraisals and feedback from significant others, particularly parents (O’Rourke et al., 2012; Schwebel et al., 2016). When athletes perceive high levels of parental support independent of performance, it reinforces global self-esteem; conversely, relational climates focused on winning make an athlete’s perceived value contingent on external success (Schwebel et al., 2016; O’Rourke et al., 2014). While global self-esteem provides the foundational psychological resources required for adaptive functioning, the transition from general self-worth to domain-specific motivation appears to require a more specialized internal mechanism (O’Rourke et al., 2014).

The transition to the specific motivational regulation required for elite performance suggests the involvement of a more specialized psychological construct: athletic identity, defined as the degree to which an individual identifies with the athlete role (Brewer et al., 1993). Stable self-esteem provides the internal resources required for a healthy, integrated identification with the athletic role, whereas a lack of foundational self-worth can lead to a rigid identification that causes distress when performance falters (Lienhart et al., 2019; Brewer and Petitpas, 2017). Importantly, developmental and sports science literatures indicate that a high identification with this role can present both benefits and risks during adolescence (Brewer and Petitpas, 2017; Park et al., 2023). Previous research shows that a restricted focus on athletic career paths can lead to identity foreclosure, which may increase vulnerability to severe depressive symptoms or emotional difficulties following unexpected injuries (Brewer and Petitpas, 2017; Park et al., 2023). Nevertheless, a well-established role-identity simultaneously offers a critical cognitive framework for competitive youth (Brewer and Petitpas, 2017). From the perspective of Self-Determination Theory (SDT), when athletes internalize their sporting involvement as a meaningful personal responsibility, this self-defining framework facilitates autonomous motivation and sport commitment (Soenens and Vansteenkiste, 2011).

Furthermore, athletic identity is closely associated with variations in autonomous motivation. According to SDT, integrated regulation occurs when an activity is fully assimilated into one’s sense of self (Deci and Ryan, 2000; Reifsteck et al., 2016). As athletes solidify their role-identity, their commitment often shifts to the internal need to act consistently with their perceived self (Jakobsson et al., 2017). This conceptual ordering is strongly supported by empirical evidence from competitive sports settings. Specifically, in a study examining elite and international-level athletes, Tušak et al. (2005) demonstrated that a highly developed athletic identity directly underpins an individual’s self-motivation and achievement orientation. These findings provide a robust empirical justification for the specified pathway, demonstrating that role-identification structurally precedes and facilitates functional motivational regulation.

The cultivation of this resilient athletic identity is also profoundly influenced by the perceived parental relationship, as parental encouragement and belief in competence validate an athlete’s emerging identity (Castillo-Jiménez et al., 2022). The integration of these perspectives suggests a complex, sequential mediation mechanism through which a positive parental relationship first solidifies foundational self-esteem, which subsequently fosters a resilient athletic identity, ultimately acting as the psychological bridge to autonomous motivation (O’Rourke et al., 2012; Reifsteck et al., 2016). Despite the documented importance of these individual variables, there is a notable paucity of research examining their collective role within a serial mediation model among elite adolescent athletes.

Therefore, the primary purpose of this study is to test a theoretically derived serial mediation model to offer preliminary evidence consistent with a proposed pathway between external familial support and autonomous motivation. By examining this sequential path, this research seeks to clarify the role of athletic identity as a domain-specific mediator that links generalized self-regard to specialized motivational regulation, while treating simple indirect effects through self-esteem or athletic identity as component tests of the broader serial model. Ultimately, this study aims to provide a clearer understanding of the pathways to optimal motivation in elite youth sports. Based on the research model, the following hypotheses are proposed:

Hypothesis 1: Perceived quality of the parental relationship will be significantly and positively associated with the autonomous motivation of elite adolescent athletes.

Hypothesis 2: Self-esteem will significantly mediate the relationship between the perceived parental relationship and autonomous motivation.

Hypothesis 3: As a component path of the serial model, athletic identity will significantly mediate the relationship between the perceived parental relationship and autonomous motivation.

Hypothesis 4: Perceived parental relationship will have a significant indirect association with autonomous motivation through the sequential mediation of self-esteem and athletic identity.

## 2 Methods

### 2.1 Participants and research procedure

The participants in this study were elite male high school soccer players officially affiliated with the Korea Football Association (KFA). To ensure the sample consisted of high-level student-athletes, a purposive sampling strategy was employed. A total of 245 athletes initially participated in the survey. During data screening, 8 participants were excluded solely because they provided identical responses across all survey items (i.e., a straight-lining pattern), indicating non-purposeful responding. Consequently, the final sample of 237 athletes was retained for analysis.

The recruitment and data collection followed a systematic ethical protocol tailored for underage athletes. Initially, the research team established contact with team directors and head coaches to secure institutional cooperation. Once authorization was granted, recruitment materials and study briefings were circulated via digital communication platforms dedicated to the athletes’ parents. This approach allowed legal guardians to autonomously evaluate the study’s risks and benefits before granting permission for their children to participate.

The survey was administered through a secure web-based interface (Google Forms). To safeguard the rights of the minors, a dual-layered electronic verification system was implemented. First, legal guardians were required to complete an informed consent module on the landing page. Only upon the guardian’s approval was the student-athlete permitted to access the formal assent section. Throughout this process, participants were explicitly informed that their involvement was entirely voluntary and that they could terminate their response at any time without any negative consequences. The entire procedure took approximately 15 to 20 min.

Regarding ethical oversight, this investigation was authorized by the Dongguk University Institutional Review Board (DUIRB-2026-04-20). Prospective participants were recruited on a purely voluntary basis, and access to the survey was granted only to those who formally acknowledged the electronic consent agreement after being debriefed on the study’s scope. All data were gathered via a confidential, anonymized platform to prevent the disclosure of any individual identities. This methodological approach ensured that the privacy rights of the student-athletes were fully protected throughout the research lifecycle.

### 2.2 Measurements

#### 2.2.1 Parental relationship

To assess the participants’ perception of their relationship with their parents, the Parental Relationship subscale from the Self-Description Questionnaire II (SDQ-II) originally developed by Marsh (1992) was employed. This subscale was developed for adolescents and has been used in many previous studies (e.g., Zheng et al., 2020; Esnaola et al., 2025); the final analysis utilized the Korean version verified by Koh (2004). Although the original scale included eight items, three items (Items 1, 3, and 5) were excluded because their standardized factor loadings were below.50 during the Confirmatory Factor Analysis (CFA), and subsequent content validity verification confirmed their inadequacy for ensuring structural integrity and model fit. Detailed information regarding the specific contents, standardized factor loadings, and reliability estimates for both the retained and excluded items is provided in Table 1. Consequently, five items (e.g., “My parents understand me,” “I like my parents”) were used for the final analysis. Each item was rated on a 5-point Likert scale ranging from 1 (Strongly Disagree) to 5 (Strongly Agree). In the present study, the Cronbach’s α for this scale was.853.

**TABLE 1:** Item refinement.

Variable	No.	Item description	β	C.R.	Status
Parental Relationship	7	Perceived parental understanding	0.872	– (Fixed)	Retained
	8	Liking one’s parents	0.821	10.981***	Retained
	4	Perceived parental respect and regard	0.778	10.502***	Retained
	2	Parental dissatisfaction or disappointment	0.672	11.476***	Retained
	6	Difficulty in communication with parents	0.623	8.642***	Retained
	5	Fairness of treatment during childhood	0.403	5.748***	Deleted
	1	Desire to raise own children like parents	0.379	5.430***	Deleted
	3	Similarity of values with parents	0.338	4.857***	Deleted
Self-Esteem	5	Having many positive feelings about self	0.838	10.340***	Retained
	4	Having strong self-confidence	0.828	10.255***	Retained
	6	Having a very good self-concept	0.823	10.214***	Retained
	2	Lacking self-confidence	0.694	9.005***	Retained
	1	Respecting oneself highly	0.649	8.543***	Retained
	7	Doing many important things in life	0.627	– (Fixed)	Retained
	3	Not respecting oneself much	0.626	8.291***	Retained
	8	Perceiving personal actions as unimportant	0.413	5.789***	Deleted
Athletic Identity	9	Spending more time thinking about sport	0.795	9.951***	Retained
	7	Having many goals related to sport	0.731	9.358***	Retained
	8	Sport being the most important part of life	0.676	8.792***	Retained
	6	Considering oneself an athlete	0.652	– (Fixed)	Retained
	10	Sport being the only important thing in life	0.528	7.119***	Retained
	5	Others seeing me primarily as an athlete	0.511	6.922***	Deleted
	3	Feeling depressed if unable to play	0.37	5.145***	Deleted
	2	Needing to play sport to feel good about self	0.27	3.809***	Deleted
	4	Most friends being athletes	0.209	2.960***	Deleted
	1	Feeling bad about self when not playing well	0.085	1.222	Deleted
Autonomous Motivation		Intrinsic Regulation	0.878	– (Fixed)	Retained
	1	Enjoyment of sports participation			
	2	Pleasure of learning sports			
	3	Satisfaction with skill improvement			
	4	Fun in discovering performance strategies			
		Integrated Regulation	0.871	17.629***	Retained
	1	Perception of sport as an absolute necessity			
	2	Importance of sport in personal life			
	3	Value and meaningfulness of sport			
	4	Sport being the core center of life			
		Identified Regulation	0.866	17.491***	Retained
	1	Acquisition of sports knowledge and skills			
	2	Desire to master difficult techniques			
	3	Personal growth and development			
	4	Advancement of overall athletic capability			

Item descriptions are phrased and condensed representations of the original scales for presentation clarity. – (Fixed) indicates the reference indicator whose factor loading was fixed to 1.0 for model identification.

***p < 0.001.

#### 2.2.2 Self-esteem

Participants’ self-esteem was measured using the General Self-Esteem subscale of the SDQ- II originally developed by Marsh (1992). This instrument was designed for adolescent populations and has been validated in prior literature; the current study implemented the Korean version verified by Koh (2004). This scale assesses the degree to which individuals respect and value themselves. While the original instrument consisted of eight items, Item 8 (“Overall, nothing I do is particularly important”) was removed following the CFA and validity check because its standardized factor loading fell below the.50 threshold, thereby improving overall factorial validity (see Table 1). The final seven items (e.g., “I have a lot of respect for myself,” “I have a lot of positive feelings about myself”) were utilized. Items were scored on a 5-point Likert scale. The Cronbach’s α for the self-esteem scale in this study was.882.

#### 2.2.3 Athletic identity

Athletic identity was measured using the Athletic Identity Measurement Scale (AIMS) developed by Brewer and Cornelius (2001) implemented via the Korean version validated for domestic adolescent and collegiate athlete populations by Park (2012). This scale evaluates the extent to which an individual identifies with the athlete role. The original 10-item scale was subjected to CFA, which led to the exclusion of five items (Items 1–5) because their standardized factor loadings were below 0.50 and subsequent content validity verification indicated that they failed to directly capture or reflect the unique athletic identity of the present elite youth sample. This *post-hoc* refinement resulted in a shortened 5-item configuration (Items 6–10) to optimize structural validity and model fit. Detailed information regarding the specific contents, standardized factor loadings, and construct reliability values for both the retained and excluded indicators is explicitly provided in Table 1. Consequently, the remaining five items (e.g., “I consider myself an athlete,” “Sport is the most important part of my life”), were used for analysis. The items were rated on a 5-point Likert scale. The Cronbach’s α for this scale was.794.

#### 2.2.4 Autonomous motivation

Autonomous motivation was assessed using the Korean version of the Sport Motivation Scale (SMS-K) validated by Lee and Jung (2017). Following the tenets of Self-Determination Theory (SDT), autonomous motivation was operationalized as a composite score of three subscales: Intrinsic Regulation (4 items; e.g., “Because I enjoy learning the sport”), Integrated Regulation (4 items; e.g., “Because sport is central to my life”), and Identified Regulation (4 items; e.g., “For my own personal development”). All 12 items were measured on a 5-point Likert scale.

The Cronbach’s α coefficients for the subscales were.913 for intrinsic regulation,.920 for integrated regulation, and.879 for identified regulation. The overall autonomous motivation factor (aggregated from the three subscales) demonstrated excellent internal consistency with a Cronbach’s α of.951.

### 2.3 Data analysis

To test the hypothesized structural relationships among the variables, data analysis was performed using a multi-step statistical approach with SPSS 26.0 and AMOS 26.0 software.

First, a measurement model analysis was conducted using Confirmatory Factor Analysis (CFA) to verify the structural integrity of the four latent constructs (Parental Relationship, Self-Esteem, Athletic Identity, and Autonomous Motivation) simultaneously. All latent variables were allowed to covary. During this stage, the model was refined by identifying and removing items with insufficient factor loadings or poor structural validity to ensure an optimal fit. Following the refinement, Cronbach’s α was calculated to assess internal consistency.

Second, based on the finalized measurement model, descriptive statistics and Pearson correlation analysis were performed to examine the general characteristics and preliminary associations between variables. To ensure the absence of multicollinearity issues before testing the structural model, a multicollinearity diagnostic was conducted by calculating Tolerance and Variance Inflation Factor (VIF) values through multiple regression analysis. Furthermore, convergent and discriminant validity were confirmed through Composite Reliability (CR) and Average Variance Extracted (AVE) values.

Third, the structural model was analyzed to test the research hypotheses. To evaluate the goodness-of-fit for both the measurement and structural models, multiple indices were utilized, including χ^2^/df, Comparative Fit Index (CFI), Tucker-Lewis Index (TLI), Root Mean Square Error of Approximation (RMSEA), and Standardized Root Mean Square Residual (SRMR).

Finally, to verify the significance of the mediation effects, a bootstrapping procedure was performed with 5,000 resamples. Notably, a phantom variable modeling approach was employed to statistically isolate and test each specific indirect effect within the serial mediation model. Bias-corrected 95% confidence intervals (CI) were calculated, where the absence of zero within the interval indicated statistical significance of the indirect effects. The statistical significance level for all analyses was set at *p* < 0.05.

## 3 Results

### 3.1 Measurement model analysis

Prior to testing the structural paths, a measurement model analysis was performed using Confirmatory Factor Analysis (CFA) to verify the psychometric reliability and validity of the measurement instruments. All four latent variables (Parental Relationship, Self-Esteem, Athletic Identity, and Autonomous Motivation) were analyzed simultaneously to evaluate the structural properties of the scales.

The evaluation indicated that the model fit indices were somewhat insufficient (χ^2^/df = 2.050, CFI = 0.872, TLI = 0.860, and RMSEA = 0.067), falling slightly below the preferred thresholds for incremental fit indices. Furthermore, an inspection of individual item properties revealed that the standardized factor loadings varied widely from 0.085 to 0.878, with multiple items presenting loadings well below the conventional conservative cutoff of 0.50. These low-loading items were identified as sources of measurement noise that compromised the convergent validity of the constructs.

To improve the validity of the scales and refine the model, an expert panel consisting of three PhDs specializing in sports psychology and measurement evaluated the content validity of the problematic items. The panel concluded that these low-loading items introduced ambiguity and response distortion, failing to accurately capture the specific psychological and athletic context of elite high school soccer players. This methodological refinement aligns with literature (e.g., Brewer and Cornelius, 2001), which demonstrated that refining the original 10-item Athletic Identity Measurement Scale (AIMS) into a shortened configuration significantly enhances model fit and measurement stability depending on the sample characteristics.

Consequently, a total of 9 items (5 from Athletic Identity, 3 from Parental Relationship, and 1 from Self-Esteem) were excluded to optimize model parsimony and measurement purity. Crucially, the expert panel verified that the remaining core items fully encompassed and represented the fundamental theoretical boundaries of each construct without compromising content validity. The complete item descriptions, operational codes, retention/exclusion status, and standardized factor loadings for all initial 29 items are comprehensively tabulated in Table 1.

After eliminating these 9 items, the finalized measurement model demonstrated a robust and improved fit to the empirical data: χ^2^/df = 2.360, CFI = 0.915, TLI = 0.902, RMSEA = 0.076, and SRMR = 0.0696. In this configuration, all standardized factor loadings were statistically significant at the *p* < 0.001 level and ranged tightly between 0.546 and 0.877, establishing strong convergent validity.

Construct reliability was supported, with Composite Reliability (CR) values ranging from 0.811 to 0.951, demonstrating high internal consistency. Convergent validity was further substantiated with Average Variance Extracted (AVE) values between 0.465 and 0.761. Although the AVE values for Self-Esteem (0.465) and Athletic Identity (0.465) fell slightly below the ideal 0.50 threshold, their respective CR values (0.882 and 0.811) comfortably exceeded the.80 level. According to Fornell and Larcker (1981), a construct’s convergent validity is deemed acceptable even with a lower AVE if its CR remains robust, validating the inclusion of the refined subscales. Lastly, discriminant validity was successfully and rigorously established based on the Fornell-Larcker criterion. As presented in Table 2, the square roots of the AVEs for all latent constructs (ranging from 0.682 to 0.872, displayed diagonally in parentheses) were consistently and significantly greater than any of the inter-construct correlation coefficients (r ranging from 0.311 to 0.619). This quantitative evidence explicitly demonstrates that the variance explained by each unique construct exceeds their shared variance with other constructs, thereby confirming the distinctiveness and psychometric integrity of the finalized measurement model.

**TABLE 2:** Descriptive statistics.

Variables	*M*	*SD*	Skewness	Kurtosis
Parental relationship	4.58	0.61	−1.69	2.17
Self-esteem	3.93	0.72	−0.29	−0.67
Athletic identity	4.42	0.60	−1.07	1.20
Autonomous motivation	4.36	0.68	−1.17	1.08
- Intrinsic regulation	4.39	0.73	−1.19	1.00
- Integrated regulation	4.42	0.73	−1.32	1.32
- Identified regulation	4.35	0.60	−0.98	1.43

### 3.2 Descriptive statistics and normality test

The descriptive statistics for the main variables Parental Relationship, Self-Esteem, Athletic Identity, and Autonomous Motivation are presented in Table 2. The mean scores for the variables ranged from 3.93 to 4.58, suggesting that the adolescent athletes in this study generally perceived a positive psychological environment and exhibited high levels of motivation.

To verify the normality of the data, skewness and kurtosis were examined. The values for skewness ranged from −1.69 to −0.29, and kurtosis values ranged from −0.67 to 2.17. According to the criteria suggested by Kline (2015), which considers skewness with an absolute value < 3.0 and kurtosis with an absolute value < 10.0 as indicative of a normal distribution, the data in this study were deemed suitable for parametric statistical analyses and structural equation modeling.

### 3.3 Correlation and multicollinearity analysis

Pearson correlation analysis was conducted to examine the preliminary relationships between the study variables (see Table 3). The results indicated that all primary variables were significantly and positively correlated with each other (*p* < 0.01). Specifically, Autonomous Motivation showed significant positive correlations with Athletic Identity (r = 0.619), Self-Esteem (r = 0.545), and Parental Relationship (r = 0.456).

**TABLE 3:** Correlation matrix.

Variables	1	2	3	4	*CR*	*AVE*
1. Parental relationship	(0.718)	(0.682)	(0.682)	(0.872)	0.853	0.516
2. Self-esteem	0.360**				0.882	0.465
3. Athletic identity	0.311**	0.526**			0.811	0.465
4. Autonomous motivation	0.456**	0.545**	0.619**		0.951	0.761

**p < 0.01. Diagonal elements in parentheses are the square roots of the AVE.

Furthermore, a multicollinearity diagnostic was performed by calculating the Tolerance and Variance Inflation Factor (VIF). As shown in the regression results, the Tolerance values (ranging from 0.681 to 0.850) were all above the threshold of.10, and the VIF values (ranging from 1.177 to 1.469) were well below the stringent criterion of 5.0. These results confirm that there are no significant multicollinearity issues among the independent variables, ensuring the stability and validity of the subsequent path analysis.

### 3.4 Structural model analysis

#### 3.4.1 Model fit and path coefficients

The structural model was tested to examine the hypothesized relationships between parental relationship, self-esteem, athletic identity, and autonomous motivation. The model fit indices indicated an acceptable fit to the data: χ^2^/df = 2.360, CFI = 0.915, TLI = 0.902, RMSEA = 0.076, and SRMR = 0.0696.

**FIGURE 1:**
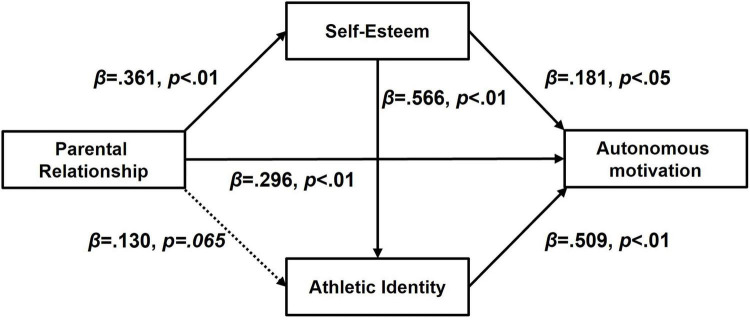
Structural path model of the research variables. Standardized coefficients (β) are presented. Solid lines indicate significant paths (*p* < 0.05), and the dashed line indicates a non-significant path (*p >* 0.05).

The results of the path analysis are summarized in Figure 1 and Table 4. Parental Relationship was significantly and positively associated with Self-Esteem (β = 0.361, *p* < 0.001) and Autonomous Motivation (β = 0.296, *p* < .001). However, its direct effect on Athletic Identity was not statistically significant (β = 0.130, *p* = 0.065), suggesting a potential full mediation through self-esteem. Self-Esteem was found to be strongly linked to Athletic Identity (β = 0.566, *p* < 0.001) and also positively related to Autonomous Motivation (β = 0.181, *p* = 0.011). Lastly, Athletic Identity exhibited a significant positive effect on Autonomous Motivation (β = 0.509, *p* < 0.001). The model accounted for 63.2% of the variance in autonomous motivation (*R*^2^ = 0.632).

**TABLE 4:** Standardized path coefficients and hypothesis testing.

Path	β	*S.E.*	*C.R. (t)*	*p*
Parental relationship → Self-esteem	0.361	0.063	4.992	*p* < 0.01
Parental relationship → Athletic identity	0.130	0.047	1.849	0.065
Self-esteem → Athletic identity	0.566	0.068	6.316	*p* < 0.01
Parental relationship → Autonomous motivation	0.296	0.059	5.163	*p* < 0.01
Self-esteem → Autonomous motivation	0.181	0.084	2.540	*p* < 0.05
Athletic identity → Autonomous motivation	0.509	0.135	5.872	*p* < 0.01

#### 3.4.2 Mediation effects analysis (bootstrapping)

To test the specific indirect effects proposed in the research model, a phantom variable modeling approach was employed. This method allows for the empirical isolation of individual indirect pathways and the calculation of their respective unstandardized coefficients, standard errors, and confidence intervals. A bootstrapping procedure with 5,000 resamples was performed, and the results of the 95% bias-corrected confidence intervals (CI) are presented in Table 5.

**TABLE 5:** Bootstrapping results for indirect effects.

Path	*B*	*S.E.*	Lower 95% CI	Upper 95% CI
Total indirect effects	0.235	0.062	0.125	0.414
Parental relationship → Self-esteem → Autonomous motivation	0.068	0.039	0.004	0.170
Parental relationship → Athletic identity → Autonomous motivation	0.068	0.048	−0.018	0.167
Parental relationship → Self-esteem → athletic identity → Autonomous motivation	0.107	0.043	0.058	0.171

The results indicate that the total indirect effect of Parental Relationship on Autonomous Motivation was statistically significant (*B* = 0.235, *S.E.* = 0.062, *p* < 0.01, 95% CI [0.125, 0.414]). Examining the specific pathways, the indirect effect of Parental Relationship on Autonomous Motivation via Self-Esteem (Hypothesis 2) was significant (*B* = 0.068, *S.E.* = 0.039, *p* = 0.035, 95% CI [0.005, 0.164]).

In contrast, the indirect effect of Parental Relationship on Autonomous Motivation through Athletic Identity (Hypothesis 3) did not reach statistical significance (*B* = 0.068, *S.E.* = 0.048, *p* = 0.133, 95% CI [−0.025, 0.169]). This finding is consistent with the non-significant direct path from Parental Relationship to Athletic Identity (*B* = 0.086, *p* = 0.065), suggesting that parental influence does not directly translate into role identification without the mediation of global self-esteem.

Finally, the sequential mediation effect of Self-Esteem and Athletic Identity in the relationship between Parental Relationship and Autonomous Motivation (Hypothesis 4) was found to be statistically significant (*B* = 0.107, *S.E*. = 0.043, *p* < 0.01, 95% CI [0.044, 0.210]). This serial pathway confirms that a positive perception of the parental relationship is first associated with an athlete’s global self-worth, which in turn may support a robust athletic identity, which in turn was associated with higher levels of autonomous motivation. These findings highlight a potential serial mechanism through which external familial support could be linked to a self-determined motivational orientation.

## 4 Discussion

The present study aimed to elucidate the psychological mechanism through which the parent-child relationship is associated with the autonomous motivation of adolescent athletes. The results of the path analysis provided meaningful insights into how perceived familial support is translated into self-determined motivational drives.

### 4.1 The direct influence of parental relationship on autonomous motivation

First, consistent with Hypothesis 1, the perception of a positive relationship with parents was found to be significantly and positively associated with autonomous motivation. This finding aligns with the fundamental tenets of Self-Determination Theory (SDT), which posits that a supportive social environment characterized by respect, emotional warmth, and mutual understanding functions as a primary antecedent for fostering a sense of volition and choice (Deci and Ryan, 2000, 2008). In the competitive ecosystem of youth sports, adolescent athletes who perceive their parents as reliable sources of emotional support are more likely to internalize the value of their athletic pursuits, shifting their motivational focus from external contingencies to personal growth and inherent enjoyment (Amorose et al., 2016; Castillo-Jiménez et al., 2022).

This result reinforces the assertion by Harwood and Knight (2015) that parents serve as the most influential socializers in an athlete’s developmental trajectory. While previous research has often focused on the role of the coach, our findings support the growing consensus that the familial environment provides the essential psychological “secure base” necessary for elite youth athletes to navigate the pressures of competitive performance (Castillo-Jiménez et al., 2022; O’Rourke et al., 2014). The positive relationship likely mitigates the perception of sports as a tool for satisfying external expectations, thereby protecting the athlete’s intrinsic drive (Lienhart et al., 2019).

Furthermore, the significant direct path from the parental relationship to autonomous motivation suggests that the qualitative nature of the parent-child bond carries intrinsic motivational value. As noted in the introduction, while excessive parental expectations can manifest as stressors (Amado et al., 2015), a relationship rooted in emotional maturity and respect (Marsh and O’Neill, 1984) acts as a catalyst for self-determined regulation. By providing organizational and emotional resources without imposing controlling pressures, parents empower athletes to perceive themselves as the primary authors of their athletic careers (Fredricks and Eccles, 2004). This support is particularly critical for adolescent athletes who are in a transitional phase of developing psychological autonomy while still remaining heavily dependent on familial structures (Wolfenden and Holt, 2005).

### 4.2 The mediating role of self-esteem between parental relationship and autonomous motivation

The results of the bootstrapping analysis confirmed that the parental relationship is indirectly linked to autonomous motivation through internal psychological processes, as evidenced by a significant total indirect effect. Specifically, consistent with Hypothesis 2, global self-esteem was found to significantly mediate the relationship between the perceived parental relationship and autonomous motivation.

This mediation pathway reinforces the theoretical perspective that an athlete’s self-worth is deeply rooted in their social environment (O’Rourke et al., 2012). For adolescent athletes, parents are not merely providers of resources but are primary evaluative mirrors. A positive and supportive parental relationship communicates an unconditional acceptance that transcends athletic success or failure, thereby fostering high levels of global self-worth (Schwebel et al., 2016). This internalized sense of being valuable as a person (Rosenberg, 1965) serves as a foundational psychological resource that allows athletes to engage in their sport with a greater sense of security and reduced anxiety regarding external validation, which is conducive to autonomous regulation (O’Rourke et al., 2014).

This finding implies that while a positive parental relationship is related to an athlete’s self-worth, global self-acceptance alone may not be the primary determinant that could be directly linked to sport-specific volition. As theorized earlier, there appears to be a conceptual gap between general self-regard and volitional action in the specific context of elite sports (Amorose et al., 2016). The transition from global self-acceptance to autonomous regulation likely requires a more specialized, domain-specific psychological bridge that aligns the activity with the individual’s internal cognitive framework (O’Rourke et al., 2014). Consequently, these results underscore the importance of exploring additional mediators, such as athletic identity, to fully understand how familial support is transformed into specialized motivational energy.

### 4.3 The non-significant mediation of athletic identity: the necessity of psychological mediation

Contrary to the initial expectation, the indirect effect of the parental relationship on autonomous motivation specifically through athletic identity (Hypothesis 3) was not statistically significant. This non-significant mediation stems from the lack of a direct predictive relationship between the perceived parental relationship and athletic identity. While prior research has emphasized that parental encouragement and belief in an athlete’s competence can validate an emerging identity (Castillo-Jiménez et al., 2022), our findings suggest that in the context of elite adolescent athletes, external familial support does not automatically translate into a robust internal role-identification.

Although parents provide the essential social signals required for identity construction (Castillo-Jiménez et al., 2022), these external appraisals may not be directly assimilated into the athlete’s self-concept unless they are first filtered through the individual’s self-worth. As theorized by Stryker and Burke (2000), role-identification is a self-regulatory mechanism that requires internal cognitive structures to maintain salience. The non-significant direct path suggests that for elite youth athletes, the mere perception of a positive relationship while motivationally beneficial is insufficient on its own to solidify a specialized identity as an athlete.

Furthermore, this finding highlights the risk of identity foreclosure if the athletic role is adopted solely based on external social validation without an integrated sense of self (Brewer and Petitpas, 2017). The fact that the direct link between parents and identity was non-significant implies that elite athletes might maintain a degree of psychological distance between their familial bonds and their athletic role-identification. This distance suggests that the development of a resilient athletic identity is a more autonomous internal process than previously assumed, requiring the mediation of global self-esteem to ensure that the athletic role is embraced as a meaningful self-expression rather than a response to familial expectations (O’Rourke et al., 2012; Lienhart et al., 2019). This underscores that external familial support does not directly dictate athletic identity; instead, it provides the emotional security needed for an athlete to voluntarily embrace their role.

### 4.4 The sequential mediating mechanism: From familial support to autonomous motivation

Finally, the most significant contribution of the present study lies in the empirical confirmation of the sequential mediation effect involving self-esteem and athletic identity (Hypothesis 4). The results demonstrated that the perceived parental relationship was significantly related to autonomous motivation through the serial pathway of self-esteem and athletic identity. This finding offers preliminary evidence consistent with a potential psychological pathway by which external social support could be linked to a self-determined motivational engine.

This sequential mechanism offers a more granular understanding of the explanatory void between generalized self-regard and domain-specific volition (Amorose et al., 2016; O’Rourke et al., 2014). As posited in the introduction, while a positive parental relationship provides the emotional security necessary for global self-esteem (Schwebel et al., 2016), this generalized sense of worth requires a specialized psychological bridge to influence an athlete’s specific regulatory orientation (Jakobsson et al., 2017). The significance of the serial path confirms that athletic identity serves as this missing psychological link, translating an athlete’s self-acceptance into a structured role-identification that directs behavior within the sporting context (Reifsteck et al., 2016).

Furthermore, the results highlight that the internalization of familial support is a tiered process. According to Identity Theory (Stryker and Burke, 2000), role-salience is maintained when an individual’s self-evaluations are consistent with their social environment. Our findings suggest that when elite adolescent athletes perceive their parents as supportive, it first stabilizes their global self-concept. This stable self-concept, in turn, provides the psychological internal resources required to embrace the demanding role of an elite athlete as a core part of their self-definition, rather than a source of pressure (Lienhart et al., 2019; O’Rourke et al., 2012). Once this athletic identity is solidified, the athlete’s motivation becomes more autonomous, as their actions are rooted in a desire to maintain consistency with their perceived self (Jakobsson et al., 2017).

While the effect sizes are relatively conservative, the statistical robustness of the serial path suggests a nuanced internalization process that simple bivariate models may overlook. Ultimately, these results provide a comprehensive, albeit initial, understanding of how the relational ecosystem of the family contributes to the cultivation of high-quality motivation in adolescent athletes.

### 4.5 Practical implications

The findings of this study offer several practical insights for parents, coaches, and sports practitioners. First, the significant serial mediation path highlights that interventions aimed at fostering autonomous motivation must prioritize the cultivation of an athlete‘s global self-esteem before focusing on role-specific identification. Parents and coaches should be educated that unconditional emotional support valuing the adolescent as a person rather than just a performer is the essential starting point for long-term motivational health. Second, the non-significant direct link from parents to athletic identity suggests that “identity-pushing” by parents may be ineffective. Instead, practitioners should encourage a familial environment that provides the psychological security necessary for athletes to voluntarily embrace and internalize their athletic identity. By focusing on the sequential internalization process identified here, sports organizations can develop more holistic support systems that promote both athletic excellence and psychological well-being.

### 4.6 Limitations and future research

Despite its contributions, the present study has several limitations that suggest directions for future research. First, the cross-sectional design of this study limits the ability to draw definitive causal inferences. Although the sequential model is grounded in established theories, longitudinal or experimental designs are needed to confirm the developmental shifts from self-esteem to athletic identity and autonomous motivation over time.

Second, the sample was limited to elite adolescent soccer players in South Korea. Given that cultural nuances regarding filial piety and parental involvement may influence these psychological pathways, future studies should include diverse sports and cultural contexts to enhance the generalizability of the findings.

Third, while the current model focused on the positive aspects of the parental relationship, future research should explore the impact of negative familial factors, such as parental pressure or over-involvement, which may function differently within the mediation mechanism. Additionally, incorporating the perspective of coaches and peers alongside parental influence would provide a more comprehensive understanding of the social ecosystem surrounding youth athletes.

Fifth, a limitation pertains to the differing conceptual levels of the variables, as parental relationships and self-esteem were assessed at a general level rather than within a sport-specific context. While examining linkages across different levels was central to our research questions, utilizing general-level constructs may have resulted in a more conservative estimation of the actual strength of these associations compared to sport-contextualized measures.

Finally, regarding the measurement properties of athletic identity, its AVE value fell slightly below the preferred threshold, and its localized indirect path was non-significant. Furthermore, the extensive *post-hoc* removal of five items from the original 10-item Athletic Identity Measurement Scale (AIMS) may have narrowed the scope of the variable, shifting its focus primarily toward identity centrality and commitment rather than broader multidimensional aspects. Consequently, this study’s findings should be interpreted with caution when making direct comparisons with previous literature that utilized the full-scale instrument. Nevertheless, these characteristics may be partly attributed to situational negative affect (e.g., competitive pressure or anxiety) inherent in elite youth populations, and the variable’s operational integrity remains robust, as its Composite Reliability comfortably exceeded the acceptable threshold, validating its empirical utility based on the Fornell-Larcker criteria.

## 5 Conclusion

The present study investigated the complex psychological pathways through which the parental relationship contributes to the autonomous motivation of elite adolescent athletes. By integrating Self-Determination Theory and Identity Theory within a serial mediation framework, this research offers preliminary evidence consistent with the internalization process of familial support.

The findings lead to several key conclusions. First, a positive and supportive parental relationship appears to function as a primary factor associated with autonomous motivation, reinforcing the critical role of parents as essential socializers in the elite sports ecosystem. Second, this influence is not merely direct but is shown to be channeled through an athlete’s global self-esteem. Specifically, self-esteem acts as a foundational resource that allows athletes to securely embrace their specialized role as an athlete. Third, the confirmation of the sequential mediation effect from parental support to self-esteem, then to athletic identity, and finally to autonomous motivation highlights that high-quality motivation may be the product of a tiered internalization process.

In conclusion, for external familial support to be effectively transformed into a self-determined motivational catalyst, it may need to first stabilize the athlete’s general sense of self-worth and subsequently solidify their domain-specific identity. These results suggest that fostering an environment that values the athlete as a whole person is highly beneficial for sustaining long-term motivational health and persistence in competitive sports.

## Data Availability

The raw data supporting the conclusions of this article will be made available by the authors, without undue reservation.
